# The Potential of LEO Satellite-Based Opportunistic Navigation for High Dynamic Applications

**DOI:** 10.3390/s22072541

**Published:** 2022-03-25

**Authors:** Nabil Jardak, Quentin Jault

**Affiliations:** French-German Research Institute of Saint-Louis, 5 Rue du Général Casssagnou, 68300 Saint-Louis, France; quentin.jault@isl.eu

**Keywords:** navigation, signals of opportunity, Doppler shift, LEO, high dynamic, INS

## Abstract

Resilient navigation in Global Navigation Satellite System (GNSS)-degraded and -denied environments is becoming more and more required for many applications. It can typically be based on multi-sensor data fusion that relies on alternative technologies to GNSS. In this work, we studied the potential of a low earth orbit (LEO) satellite communication system for a high-dynamic application, when it is integrated with an inertial measurement unit (IMU) and magnetometers. We derived the influence of the main error sources that affect the LEO space vehicle (SV) Doppler-based navigation on both positioning and attitude estimations. This allowed us to determine the best, intermediate and worst cases of navigation performances. We show that while the positioning error is large due to large orbit errors or high SV clock drifts, it becomes competitive with that of an inertial navigation system (INS) based on a better quality IMU if precise satellite orbits are available. On the other hand, the attitude estimation tolerates large orbit errors and high SV clock drifts. The obtained results suggest that LEO SV signals, used as signals of opportunity for navigation, are an attractive alternative in GNSS-denied environments for high dynamic vehicles.

## 1. Introduction

High dynamic vehicles require resilient navigation solutions. Indeed, the Global Navigation Satellite System (GNSS), which is the leading technology of positioning navigation and timing, is sensitive to jamming and spoofing. To cope with such a situation, the GNSS community has developed interference mitigation techniques, but another complementary solution is that which uses an alternative technology to GNSS when it becomes inoperative.

This work deals with the navigation using signals of opportunity (SOP), more specifically the signals of the Iridium Next Communication System. SOP are all radio frequency (RF) signals that are not intended for navigation purposes. They are signals of communication systems (mobile networks, satellite-based communication systems), TVs, AM/FM radios, radar, etc. The advantages of SOP in navigation are the existing infrastructure that is free of use, the high signal power level compared to that of GNSS signals, and the high frequency diversity if many systems are used. The disadvantages of SOP stem from the fact that the signals are not optimized for navigation purposes. That is, the signals’ availability is not guaranteed everywhere, signals from different transmitters (including those of the same system) are not synchronized in time and their clock stability is lower than that of GNSS satellites, and transmitter positions are unknown. In addition, if different systems are coupled, one needs multi-band antennas, multi-band RF front-end and a sufficient computing power.

Despite these constraints, finding an alternative or an augmentation technology to GNSS is crucial. The research in the topic of navigation based on SOP essentially started two decades ago, and has gained interest over the last few years, especially with the announcement of the advent of future new mega LEO satellite (LEO SV) constellations, Starlink and OneWeb, and the modernization of the Iridium constellation (which became Iridium Next in 2017). The popularity of LEO SV systems for navigation is mainly thanks to their global coverage not only on Earth, but also in its surrounding space, which allows high-altitude vehicles to be potential users.

In this paper, we study the potential of the Iridium Next LEO SV signals for the navigation of a high dynamic vehicle. The Iridium Next system offers voice and data communication. The company Satelles used the messaging to transmit bursts designed specifically for the navigation in a PNT solution called Satellite Time and Location (STL) [1]. Since positioning and namely timing capabilities have been demonstrated based on the STL technology, in this work, we will use the Iridium Next signals as signals of opportunity—that is, no navigation specific data are extracted from the signal in space.

Navigation using LEO SV signals as SOP has been the focus of many papers. In [2], a differential positioning using time difference of arrival (TDOA) and frequency difference of arrival (FDOA) is implemented. Experiments showed a positioning accuracy of 25 m with 5 min data collected in a static location. In [3], the authors studied a positioning using LEO SV Doppler shifts that are loosely fused with an altimeter height. Experiments, using 1 min data from two Orbcomm satellites and by assuming a known altitude, exhibited a 2D positioning error of 358 m for a static observer. In [4,5], the authors derived the performance of LEO SV measurements fused with an inertial navigation system (INS). A range of information from LEO SVs is assumed to be available in addition to the Doppler shifts. The algorithm refines the satellite position and velocity estimates by including them into the state in addition to satellite clock biases and drifts. The global positioning system (GPS) is only used at the beginning, and then the navigation filter is updated by LEO SV and altimeter height measurements. An experiment using 4 min data of 2 Orbcomm SV led to a positioning accuracy with a root mean square error (RMSE) of a few hundred meters for a land vehicle. In [6], positioning using Iridium signals in forest canopy is demonstrated. Experiments showed a height aided static positioning accuracy of a few hundred meters obtained with 30 min data. In [7], the authors showed that the satellite position accuracy, based on the Simplified Perturbation model (SPG4) fed with Two Line Element (TLE) files, can be as high as 3 km, and the velocity accuracy can be as high as 3 m/s. They showed that the two-body model using the second gravitational zonal coefficient J2 allows for a better satellite positioning, if a good initial satellite position is available. In [8], the authors presented a Doppler-based positioning using Ku-band Starlink signals. A 3D positioning better than 23 m was shown by the experiment in which the TLE epochs were adjusted to cope with the ephemeris errors. In [9], a Multi-Constellation Software-Defined Receiver was designed to measure the Doppler shifts of LEO SV downlink signals. The paper showed by means of the experiment the benefits of the multi-constellation (Orbcomm and Iridium systems) for the positioning. A multi-constellation LEO SV signal receiver was also presented in [10]. The demonstrated positioning accuracy is 22.7 m, obtained by assuming a known altitude of the user, and when up to four LEO SVs were tracked from Iridium and Orbcomm systems. In [11], the Doppler shift is measured on signals with orthogonal frequency-division multiplexing (OFDM) modulation, which is expected for the deployment of future LEO SV. Experiments using new 5G terrestrial radio signals showed an RMSE of 6.45 Hz in the estimation of the Doppler shift. In [12], a carrier phase-based positioning on LEO SV signals was designed. Then a 3D positioning accuracy of 33.5 m was achieved by the experiment using six Starlink SV signals.

The synthesis of this existing work shows that the navigation is typically performed based on the Doppler shift measurement, which is a straightforward observable that can be obtained from a radio signal transmitter with a known carrier frequency. A first category of LEO SV-based navigation techniques uses only LEO SV signals. While the positioning is usually standalone, the differential mode involving two observers has also been explored. A second category performs the navigation based on the coupling between LEO SV and an INS (based on gyroscopes and accelerometers). In general, the tight coupling is considered due to the poor availability of the LEO SVs. In both categories, the navigation is either single- or multi-constellation (involving two or more LEO SV systems) and is eventually height-aided.

These developments and similar work [13,14,15,16] demonstrated the potential of LEO SV signals for the opportunistic positioning. In this work, we will address the following complementary tasks

- Evaluate the potential of LEO SV SOP for a high dynamic vehicle with a short-duration mission. The short duration is expected to prevent achieving an optimal navigation performance.
- Design a filter measurement model based on magnetometer outputs and LEO SV Doppler shift that allows for observing the position, velocity, attitude, and receiver clock bias and drift.
- Use of representative Iridium data for performance simulation. Indeed, the Doppler shift measurements are obtained by processing Iridium-like signals by an Iridium signal-processing tool. These Doppler shift data are more representative than those obtained by simply adding an error distribution to the geometric Doppler shift between a satellite and a user terminal.
- Evaluate the performance of attitude estimation in addition to that of positioning. Note that the attitude and especially the roll angle can be used to roll-rate stabilize the vehicle, and in guidance [17].
- Evaluate the influence of the individual error sources on Doppler-based navigation. The errors consist mainly of the satellite position and velocity errors, the satellite clock drift, and thermal noise.
- Benchmark the performance of the system against an INS based on a tactical-grade IMU and magnetometers.

This paper is organized as follows. After this introductive section (Section 1), Section 2 defines the tight coupling filter between IMU, LEO SV Doppler shift and magnetometer data. Section 3 presents the simulation and signal processing tools developed to conduct this study. Section 4 studies the performance of the positioning and the attitude estimation in which the effect of each error source is determined, and the performance in a typical intermediate case and worst case are derived and compared to an INS solution. Section 5 concludes the paper.

## 2. The Navigation Filter

**Figure 1:**
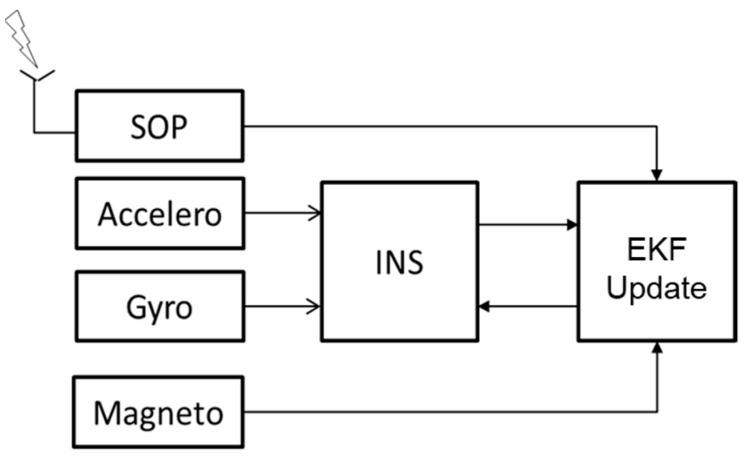
The principle of the INS/SOP tight coupling.

The navigation filter implements a tight coupling between IMU, the Iridium Doppler shift and the magnetometer (Figure 1). Here, we take advantage of the complementarity between the inertial navigation that has a good performance in the short term but diverges with time, and the Doppler measurements from Iridium satellites that are very noisy in the short term but should be relatively stable at the long term. This algorithm is different from the usual GNSS/IMU tight coupling in three aspects: (1) it uses only LEO SV Doppler shift measurements, not pseudoranges; (2) the measurements from satellites in view over the same period of time come at different instances as the corresponding bursts do, and thus the innovation at each time update contains usually a single Doppler shift measurement, in addition to the Earth’s magnetic field measurements; and (3) the use of magnetometers for a direct attitude observation, especially when dealing with a spinning vehicle.

**Figure 2:**
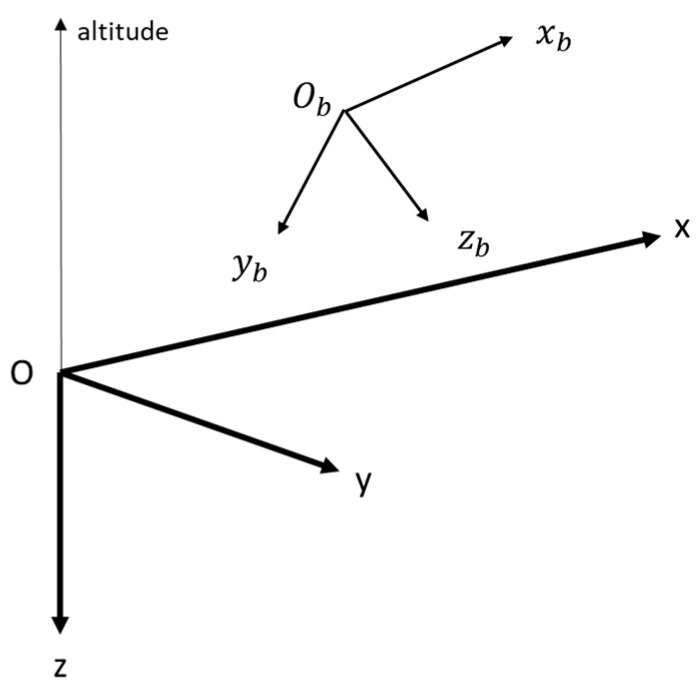
The local frame (*O*, *x*, *y*, *z*) and the body frame ($O_{b}$,$x_{b}$,$y_{b}$,$\;z_{b}$ ).

We define the local frame (*O*, *x*, *y*, *z*) such that the *x-y* plane is tangent to the Earth ellipsoid at point *O*, with (*Ox*) towards a given direction (the geographic north for instance), (*Oz*) is the vertical downward direction and (*Oy*) completes the direct coordinate system. The body frame ($O_{b}$, $x_{b}$, $y_{b}$, $z_{b}$) is centred on the vehicle’s center of mass, with ($O_{b}x_{b}$) being the vehicle longitudinal axis oriented towards the movement direction, and the $y_{b}$-$z_{b}$ plane being perpendicular to ($O_{b}x_{b}$). Both frames are visible in Figure 2. The attitude angles (roll: ϕ, pitch: θ, yaw: ψ) are defined as the angles that allow the transformation between the body frame and the local frame. The three-axis accelerometer, the three-axis gyroscope and the three-axis magnetometer are supposed to be aligned with the body frame axes. The L1 band (1.626 GHz) antenna is supposed to be located at $O_{b}$. These assumptions are intended to simplify the filter model.

The navigation algorithm implements an extended Kalman filter (EKF). The state vector is formed by the position error $\delta r=\hat{r}-r$ and the velocity error $\delta v=\hat{v}-v$**,** both in the navigation frame, the attitude error $\delta \epsilon =\hat{\epsilon }-\epsilon$, the accelerometer biases error $\delta b_{a}={\hat{b}}_{a}-b_{a}$ and the gyroscope biases error $\delta b_{g}={\hat{b}}_{g}-b_{g}$, both in the body frame, the receiver clock bias error $\delta \left(\delta t\right)=\hat{\delta t}-\delta t$ and the receiver clock drift error $\delta \left(\dot{\delta t}\right)=\hat{\dot{\delta t}}-\dot{\delta t}$ converted in m and in m/s, respectively:

(1) $$\delta x={\left[\delta r,\delta v,\delta \epsilon ,\delta b_{a},\delta b_{g},\delta \left(c\delta t\right),\delta \left(c\dot{\delta t}\right)\right]}^{T}$$

c is the signal propagation speed.

### 2.1. Dynamic Model

The filter dynamics can be written as [18]:

(2) $$\begin{array}{c} \delta \dot{r}\;=\delta v\\ \delta \dot{\epsilon }=R_{b}^{l}\delta b_{g}+R_{b}^{l}\eta_{g}\\ \delta \dot{v}=-\left(R_{b}^{l}{\tilde{f}}^{b}\times \right)\delta \epsilon +R_{b}^{l}\delta b_{a}+R_{b}^{l}\eta_{a}\\ \delta \dot{b_{a}}=-\tau_{a}^{-1}\delta b_{a}+w_{a}\\ \delta \dot{b_{g}}=-\tau_{g}^{-1}\delta b_{g}+w_{g}\\ \dot{\delta \left(c\delta t\right)}=\delta \left(c\dot{\delta t}\right)+w_{b}\\ \delta \left(c\ddot{\delta t}\right)=w_{d} \end{array}$$

where $R_{b}^{l}$ is the matrix that allows the transformation between the body frame and the local frame.

(3) $$R_{b}^{l}=\left(\begin{array}{c} c\phi \;c\psi +s\phi \;s\theta \;s\psi \\ -c\phi \;s\psi +s\phi \;s\theta \;c\psi \\ -s\phi \;c\theta \end{array}\;\;\begin{array}{c} c\theta \;s\psi \\ c\theta \;c\psi \\ s\theta \end{array}\;\;\begin{array}{c} s\phi \;c\psi -c\phi \;s\theta \;s\psi \\ -s\phi \;s\psi -c\phi \;s\theta \;c\psi \\ c\phi \;c\theta \end{array}\right)$$

where c and s are the cosine and sine functions, respectively.

${\tilde{f}}^{b}$ is the specific force measured by the accelerometer and corrected for the accelerometer biases ${\hat{b}}_{a}$. (.×) is the skew-symmetric matrix operator. $\eta_{a}$ and $\eta_{g}$ are, respectively, the accelerometer and gyroscope noise processes. Sensor biases are modelled by a first order Gauss–Markov process with correlation times $\tau_{a}$ and $\tau_{g}$ and noises $w_{a}$ and $w_{g}$, respectively, for the accelerometer and the gyroscope. The time derivative of the receiver clock bias error equals the clock drift error plus a Gaussian noise $w_{b}$. The receiver clock drift error is a constant random process, and its time derivative is a Gaussian noise $w_{d}$.

The system defined in (2) can be written as $\dot{\delta x}=F\delta x+W$, with F being the dynamic matrix and W being the system noise matrix with covariance Q=cov(W). The attitude is predicted by the integration of the differential equation ${\dot{R}}_{b}^{l}≅R_{b}^{l}\left(\omega^{b}\times \right)$, where $\omega^{b}\;$= $\left({\tilde{\omega }}^{b}-{\hat{b}}_{g}\right)$ is the angular velocity vector in the body frame provided by the gyroscope and corrected for the gyroscope biases. The user acceleration in the local frame is obtained by $a={\hat{R}}_{b}^{l}\left({\tilde{f}}^{b}-{\hat{b}}_{a}\right)+g$, with g being the local gravity vector. The receiver clock bias δt is predicted by the integration of the receiver clock drift $\dot{\delta t}$. The velocity in the local frame v is obtained by the integration of the acceleration a. Then, the position r in the local frame is obtained by the integration of the velocity v. Finally, the state covariance matrix P is computed according to $P=\Phi P\Phi^{T}+\Delta tQ$*,* where Φ=I+ΔtF is the state transition matrix and Δt is the time step of the prediction.

### 2.2. Observation Model

The measurements consist of the Doppler shifts from LEO SVs (converted to range rates) and the Earth’s magnetic field from a triad of magnetometers.

#### 2.2.1. Doppler Shift Model

The Doppler shift measurement is subjected to many error sources. The Multipath error is negligible for a vehicle travelling in an open sky. The Doppler shift induced by the ionosphere delay variation is higher than that of GNSS due to the faster variation of the ionosphere pierce point of the LEO SVs. Nevertheless, simulations we carried out using ionosphere grid maps showed that the ionosphere-induced Doppler shift stays largely below 1 Hz at 1.626 GHz. The tropospheric delay variation is dominated by the fast height variation of the vehicle rather than the elevation variation of the satellite. The usage of a tropospheric delay correction model (for instance, the MOPS [19]) is known to compensate accurately for this error. The expected residual is therefore small enough compared to the Doppler shift measurement noise. In this study, we will ignore the Doppler shift induced by the atmosphere and by the multipath propagation.

The *k*th-satellite range rate measurement, ${\dot{\rho }}^{k}$ (in m/s), is given by the opposite sign of the Doppler frequency shift $F_{d}$ multiplied by the carrier wavelength λ. It can be written as the relative velocity between the receiver’s antenna and the transmitter’s antenna projected onto the line of sight (LOS) unit vector $u^{k}$ between them, plus the receiver clock drift (converted in m/s), $c\dot{\delta t}$, minus the satellite clock drift, $c\dot{\delta t^{k}}$ (converted in m/s), and an additive Gaussian thermal noise $\eta_{\dot{\rho }}$ [20]

(4) $${\dot{\rho }}^{k}=-\lambda F_{d}≅{\left(u^{k}\right)}^{T}\left(v-v^{k}\right)+c\dot{\delta t}-c\dot{\delta t^{k}}+\eta_{\dot{\rho }}$$

The LOS unit vector $u^{k}$ is written as:

(5) $$u^{k}=\frac{r-r^{k}}{\left\|r-r^{k}\right\|}$$

where $r^{k}=r^{k}\left(t-\tau^{k}\right)$ and $v^{k}=v^{k}\left(t-\tau^{k}\right)$ are, respectively, the position and the velocity of the *k*th-satellite at the time of burst transmission $t-\tau^{k}$, where t is the burst reception time and $\tau^{k}$ is the propagation delay of the signal between the satellite antenna and the receiver antenna.

Usually, if the satellite clock drift $\dot{\delta t^{k}}$ is known, it is used to correct the range rate measurement ${\dot{\rho }}^{k}$. In our case, it is unavailable. The predicted range rate computed at the burst reception time can be written as:

(6) $${\hat{\dot{\rho }}}^{k}={\left(\frac{\hat{r}-r^{k}\left(\hat{t}-\tau^{k}\right)}{\left\|\hat{r}-r^{k}\left(\hat{t}-\tau^{k}\right)\right\|}\right)}^{T}\left(\hat{v}-v^{k}\left(\hat{t}-\tau^{k}\right)\right)+c\hat{\dot{\delta t}}$$

In (6), we assumed that the reconstructed propagation time is close to the actual propagation time once the filter has converged, i.e., ${\hat{\tau }}^{k}\approx \tau^{k}$.

In order to linearize the range rate model, we define the following perturbations of the user position, user velocity, user time and user clock drift, respectively:

(7) $$\begin{array}{c} \hat{r}=r+\delta r\\ \hat{v}=v+\delta v\\ \hat{t}=t+\delta t\\ \hat{\dot{\delta t}}=\dot{\delta t}+\delta \left(\dot{\delta t}\right) \end{array}$$

By inserting (7) into (6), we obtain

(8) $${\hat{\dot{\rho }}}^{k}=\frac{{\left(r+\delta r-r^{k}\left(t+\delta t-\tau^{k}\right)\right)}^{T}}{\left\|r+\delta r-r^{k}\left(t+\delta t-\tau^{k}\right)\right\|}\left(v+\delta v-v^{k}\left(t+\delta t-\tau^{k}\right)\right)+c\dot{\delta t}+\delta \left(c\dot{\delta t}\right)$$

The satellite position and velocity at the biased emission time can be written as:

(9) $$r^{k}\left(t+\delta t-\tau^{k}\right)=r^{k}\left(t-\tau^{k}\right)+\delta t{\dot{r}}^{k}\left(t-\tau^{k}\right)$$

(10) $$v^{k}\left(t+\delta t-\tau^{k}\right)=v^{k}\left(t-\tau^{k}\right)+\delta t{\dot{v}}^{k}\left(t-\tau^{k}\right)$$

Inserting (9) and (10) into (8) yields

(11) $${\hat{\dot{\rho }}}^{k}=\frac{{\left(r-r^{k}+\delta r-\delta t{\dot{r}}^{k}\right)}^{T}}{\left\|r-r^{k}+\delta r-\delta t{\dot{r}}^{k}\right\|}\left(v-v^{k}+\delta v-\delta t{\dot{v}}^{k}\right)+c\dot{\delta t}+\delta \left(c\dot{\delta t}\right)$$

In (11), the reference to time $t-\tau^{k}$ in $r^{k}$, ${\dot{r}}^{k}$,$\;v^{k}$, and ${\dot{v}}^{k}$ is omitted for clarity. To develop the predicted range rate (11), we can write

(12) $$\frac{1}{\left\|r-r^{k}+\delta r-\delta t{\dot{r}}^{k}\right\|}≅\frac{1}{\left\|r-r^{k}\right\|}\left(1-\frac{{\left(r-r_{k}\right)}^{T}\delta r}{{\left\|r-r^{k}\right\|}^{2}}+\frac{{\left(r-r_{k}\right)}^{T}{\dot{r}}^{k}}{{\left\|r-r^{k}\right\|}^{2}}\delta t\right)$$

In (12), we neglected the quadratic terms (i.e., $\delta t^{2}{\left\|{\dot{r}}^{k}\right\|}^{2}\approx 0,$ ${\left\|\delta r\right\|}^{2}\approx 0,$ and $\delta t\delta r^{T}{\dot{r}}^{k}\approx 0$) and we limited the development to the first order for small values of δr and δt.

Substituting $\frac{r-r^{k}}{\left\|r-r^{k}\right\|}$ by $u^{k}$ in (12), and then inserting (12) into (11), we obtain the following approximation of the range rate

(13) $$\begin{array}{cc} {\hat{\dot{\rho }}}^{k}≅ & \frac{{\left(r-r^{k}+\delta r-\delta t{\dot{r}}^{k}\right)}^{T}}{\left\|r-r^{k}\right\|}\left(1-\frac{{u^{k}}^{T}\delta r}{\left\|r-r^{k}\right\|}+\frac{{u^{k}}^{T}{\dot{r}}^{k}}{\left\|r-r^{k}\right\|}\delta t\right)\left(v-v^{k}+\delta v-\delta t{\dot{v}}^{k}\right)\\ & +c\dot{\delta t}+\delta \left(c\dot{\delta t}\right) \end{array}$$

The development of (13) by neglecting the second order terms yields

(14) $$\begin{array}{c} {\hat{\dot{\rho }}}^{k}≅{\dot{\rho }}^{k}+\left(\frac{{\left(v-v^{k}\right)}^{T}}{\left\|r-r^{k}\right\|}-{u^{k}}^{T}\left(v-v^{k}\right)\frac{{u^{k}}^{T}}{\left\|r-r^{k}\right\|}\;\right)\delta r+{u^{k}}^{T}\delta v+\\ \left(\frac{\left({u^{k}}^{T}{\dot{r}}^{k}\right){u^{k}}^{T}-{{\dot{r}}^{k}}^{T}}{\left\|r-r^{k}\right\|}\left(v-v^{k}\right)-{u^{k}}^{T}{\dot{v}}^{k}\right)\delta t+\delta \left(c\dot{\delta t}\right)+\eta_{\dot{\rho }} \end{array}$$

Therefore, the range rate error defined by $\Delta {\dot{\rho }}^{k}={\hat{\dot{\rho }}}^{k}-{\dot{\rho }}^{k}$ can be written as:

(15) $$\begin{array}{c} \Delta {\dot{\rho }}^{k}≅\left(\frac{{\left(v-v^{k}\right)}^{T}}{\left\|r-r^{k}\right\|}-{u^{k}}^{T}\left(v-v^{k}\right)\frac{{u^{k}}^{T}}{\left\|r-r^{k}\right\|}\;\right)\delta r+{u^{k}}^{T}\delta v+\\ \frac{1}{c}\left(\frac{\left({u^{k}}^{T}{\dot{r}}^{k}\right){u^{k}}^{T}-{{\dot{r}}^{k}}^{T}}{\left\|r-r^{k}\right\|}\left(v-v^{k}\right)-{u^{k}}^{T}{\dot{v}}^{k}\right)c\delta t+\delta \left(c\dot{\delta t}\right)+\eta_{\dot{\rho }} \end{array}$$

Finally, the range rate error of (15) can be written in the following form:

(16) $$\Delta {\dot{\rho }}^{k}≅h_{r}\;\delta r+h_{v}\;\delta v+h_{c\delta t}\;c\delta t+\delta \left(c\dot{\delta t}\right)+\eta_{\dot{\rho }}$$

where the coefficients $h_{r}$, $h_{v}$ and $h_{c\delta t}$ can be easily deduced by identification between (15) and (16).

#### 2.2.2. Magnetic Field Measurement Model

The earth’s magnetic field in the local frame is obtained from the magnetometer measurement ${\tilde{B}}^{b}$ as follows

(17) $${\tilde{B}}^{l}={\hat{R}}_{b}^{l}{\tilde{B}}^{b}$$

The magnetometer is supposed to be calibrated so that the measurement error is dominated by the sensor noise $\eta_{B}$ as

(18) $${\tilde{B}}^{b}=B^{b}+\eta_{B}$$

We chose this model for the sake of simplicity. Note that the Earth’s magnetic field measured by a magnetometer is distorted, in addition to the sensor errors and their misalignment with the body frame axes, by the perturbations due to the ferromagnetic material composing the vehicle and by the current induced by the rotation of the metallic vehicle. In practice, those errors can be calibrated [21,22,23,24] and the residuals will add to the sensors bias and scale factor errors. A model that includes the magnetometer bias and scale factor in the filter can be found in [25].

For small attitude errors δϵ, ${\hat{R}}_{b}^{l}$ can be written as:

(19) $${\hat{R}}_{b}^{l}=\left(I_{3}-\left(\delta \epsilon \times \right)\right)R_{b}^{l}$$

Inserting (18) and (19) into (17) yields

(20) $${\tilde{B}}^{l}=B^{l}+\left({\tilde{B}}^{l}\times \right)\delta \epsilon +R_{b}^{l}\eta_{B}$$

The earth’s magnetic field error in the local frame $\Delta B^{l}={\tilde{B}}^{l}-B^{l}$ is therefore

(21) $$\Delta B^{l}=\left({\tilde{B}}^{l}\times \right)\delta \epsilon +R_{b}^{l}\eta_{B}$$

The reference Earth’s magnetic field in the local frame, $B^{l}$**,** can be obtained for instance from the World Magnetic Model [26].

#### 2.2.3. Update

Equations (16) and (21) allow us to express the observation errors $\Delta z={\left[\Delta {\dot{\rho }}^{k},\;\Delta B^{l}\right]}^{T}$ as a linear function of the error state δx as

(22) $$\Delta z≅H\delta x+\eta_{z}$$

In (22), H is the observation model matrix given by

(23) $$H=\left(\begin{array}{cc} h_{r} & h_{v}\\ 0_{3,3} & 0_{3,3} \end{array}\;\;\;\begin{array}{cc} 0_{1,3} & 0_{1,3}\\ \left({\tilde{B}}^{l}\times \right) & 0_{3,3} \end{array}\;\;\begin{array}{cc} 0_{1,3} & h_{c\delta t}\\ 0_{3,3} & 0_{3,1} \end{array}\;\;\begin{array}{c} 1\\ 0_{3,1} \end{array}\right)$$

and $\eta_{z}$ is the observation model noise given by

(24) $$\eta_{z}={\left[\eta_{\dot{\rho }},R_{b}^{l}\eta_{B}\right]}^{T}$$

The noise covariance, $R=cov\left(\eta_{z}\right)$, is a diagonal matrix formed by the variance of the Doppler shift noise and the variances of the three-axis magnetometer noises.

The predicted state x and the covariance matrix P are then updated as follows:

(25) $$\begin{array}{c} x=x+K\Delta z\\ P=P-KHP \end{array}$$

where K  is the EKF gain given by $K=PH^{T}{\left(HPH^{T}+R\right)}^{-1}.$

## 3. Simulation and Signal Processing Tools

We developed simulation and signal processing tools in order to support the study of the potential of LEO SV signals for the navigation of a high dynamic vehicle.

### 3.1. Iridium Signal Processing Tool

Iridium Next downlink signals come in discontinued bursts transmitted by 75 satellites that orbit the Earth at an altitude of 780 km. A downlink burst is transmitted in the band 1616–1626.5 MHz using one of the several frequency channels available following the FDMA multiple access. The system has simplex and duplex channels; we used the simplex channels (1626–1626.5 MHz) because they are broadcast regularly worldwide on fixed carrier frequencies (the ring alert and the messaging channels). Bursts have a common structure composed of a tone over $T_{tone}$ = 2.56 ms, followed by a BPSK word over a short period, then followed by QPSK data until the end of the burst. The data rate is Rs = 25 ksps. The burst duration of a simplex channel does not exceed $T_{burst}\leq$ 20.32 ms [6,27].

**Figure 3:**
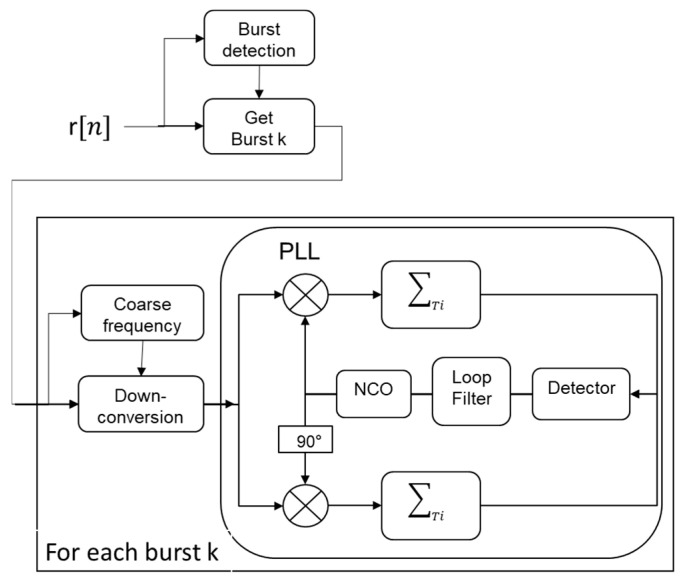
Block diagram of the Iridium signal processing tool.

Based on the Iridium signal structure, we designed an Iridium signal processing tool to detect Iridium bursts and measure their carrier frequencies. The tool shown in Figure 3 works as follows.

We perform burst detection by comparing the power level of the incoming signal r to a threshold T that is supposed to represent the noise power level (i.e., without the useful signal)

(26) $$\tilde{y}\left[n\right]=N^{-1}\sum_{i=0}^{N-1}{\left|r\left[n-i\right]\right|}^{2}\;\;\;\;⪑\;\;\;T$$

where *N* is the size of the averaging window. When the signal average power exceeds the threshold, the burst start epoch $k_{start}$ and the burst end epoch $k_{end}$ are determined. Then, for each detected burst $s=r\left[k_{start}:k_{end}\right]$, we compute the spectrum of the burst tone. The frequency of the maximum of the spectrum yields a coarse estimation of the burst carrier frequency ${\hat{f}}_{c}$ as

(27) $${\hat{f}}_{c}=argmax_{f}\left\{\;DFT\left(r\left[k_{start}:k_{start}+T_{tone}F_{s}\right]\right)\right\}$$

where DFT(.) is the discrete Fourier transform and $F_{s}$ is the sampling frequency. We then use this approximate frequency to down-convert the burst s to baseband

(28) $$s_{bb}=s\times exp\left(j2\pi \left({\hat{f}}_{c}t\right)\right),\;\mathrm{with}\;t=\left[0:\frac{1}{F_{s}}:T_{burst}\right]$$

Finally, the baseband burst $s_{bb}\;$enters a phase lock loop (PLL) where its carrier frequency estimation is refined. The PLL is composed of a numerical controlled oscillator (NCO), an integrate-and-dump over *Ti*, $\underset{Ti}{\sum }\left(.\right)$, an arctangent phase detector and a first-order loop filter. At this stage, the Doppler frequency is given by the difference between the carrier frequency estimated by the PLL and the transmitter frequency.

**Figure 4:**
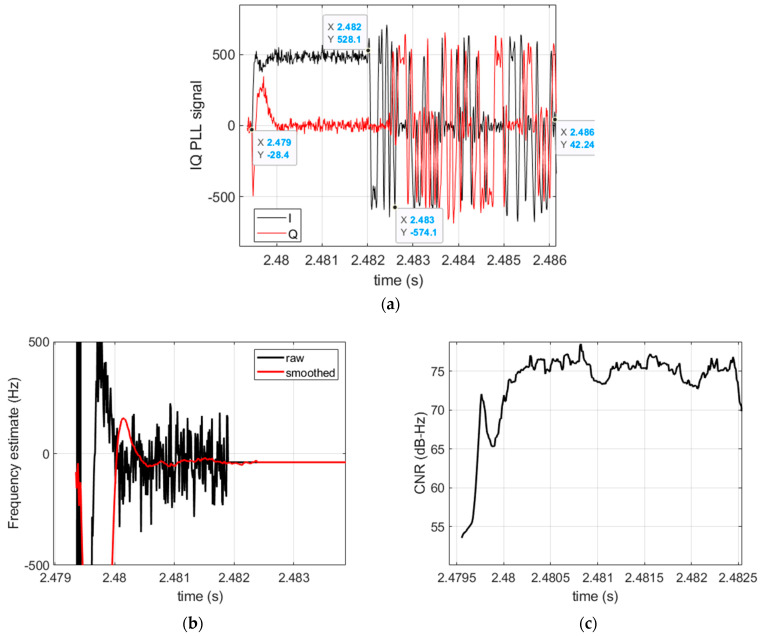
Processing results of an Iridium burst. (**a**) Baseband signal showing the structure of the burst consisting of a tone over ~2.5 ms, followed by a BPSK sequence, and completed by a QPSK sequence; (**b**) the PLL frequency estimate and its filtered value; (**c**) CNR estimate.

We processed real life Iridium signals captured in a static location by means of a USRP-N310. We used an active right hand circular polarized antenna with a ~37 dB gain in the Iridium frequency band. The antenna was located on the roof of our building to maximize signal reception. This solution provides Iridium bursts and their Doppler shift measurements with a timestamp capability better than 1 s. The digitizer has been configured with a sampling frequency of 2 MHz around Fo = 1626.25 MHz. The processing tool is configured as follows. During the burst detection, the average signal power is computed over a duration of 1 ms. The PLL coherent integration time Ti is set to 10 µs, the PLL equivalent noise bandwidth Bn is set such that Bn×Ti = 0.02. Figure 4 illustrates the processing results of a single burst. As bursts are very short, the PLL requires a large loop bandwidth and thus a small integration time that leads to high noise frequency estimates.

**Figure 5:**
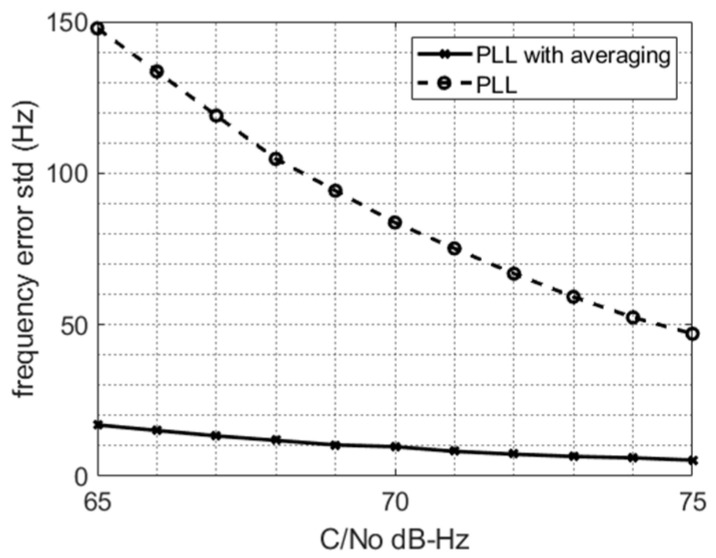
PLL frequency thermal noise std along with the std of the averaged frequency estimate over *Ta* = 50 × *Ti*.

In order to improve the frequency precision, we averaged the frequency estimated by the PLL over time. Figure 5 shows the expected PLL frequency thermal noise standard deviation (std) as a function of the carrier-to-noise density ratio (CNR) as well as the frequency thermal noise std obtained after averaging over the last *Ta* = 0.5 ms of the burst tone. Such an averaging reduced the frequency error spread by a factor of $\sqrt{Ta/Ti}\;$~7.

**Figure 6:**
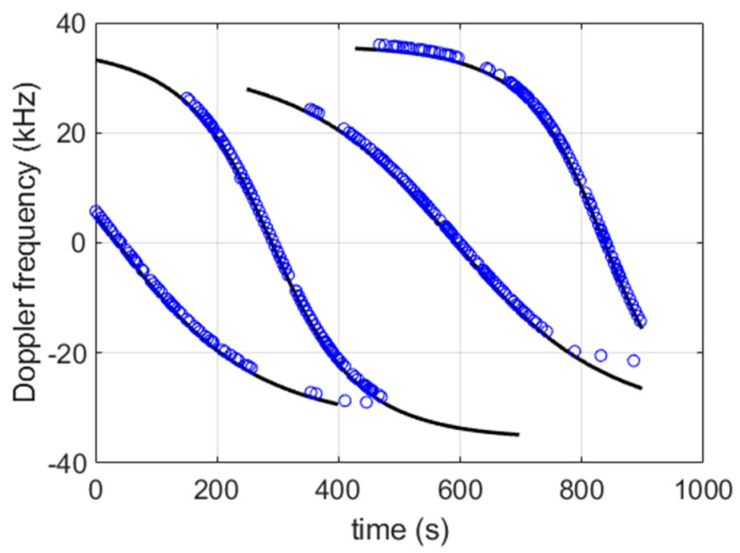
Estimated Doppler frequency (blue circles) along with expectation (black curves). From left to right: SV number 135, 167, 151, and 171.

In Figure 6, we compare the Doppler shift measurements of real bursts to the expected values computed based on the satellite orbits (TLE file of the day of the collect [28]), the position of the observer (static), and the signal recording timestamp. The results show that the measurements and the expectations are consistent. The residual difference can be explained by:

- the approximate satellites orbits based on TLE-SPG4 used to compute the expected Doppler shift,
- the timestamp error of the records, which is better than 1 s (a time error leads to satellite position and velocity errors, which convert to an error in the expected Doppler shift),
- the clock drifts of the satellites and the signal recorder, which are not taken into account in the expected Doppler shift calculation.

We also noticed the periods where we do not have measurements (namely at the beginning when a satellite appears or at the end when it disappears). This is because of the weak signal strength at these periods.

### 3.2. Iridium-like Signal Simulator

We developed an Iridium-like signal simulator software that consists of a constellator, a waveform generator and a signal conditioner. The constellator generates the ranging and power information for each satellite visible to the observer. The satellites positions and velocities are computed at times of bursts transmission based on the SPG4 model fed with a TLE file (this latter contains the Iridium Next satellite orbit parameters diffused by NORAD). They are then used together with a user-defined trajectory to compute at each epoch t the range ρ(t) (distance between the satellite’s and the receiver’s antennas), the range rate $\dot{\rho }\left(t\right)$ and the received signal power level P(t) for the satellites in view. The range and range rate are perturbed to take into account the receiver and satellites clock errors as:

(29) $${\tilde{\rho }}^{k}=\rho^{k}+c\;\delta t-c\delta t^{k}\;\;\left[m\right]$$

(30) $${\dot{\tilde{\rho }}}^{k}={\dot{\rho }}^{k}+c\;\dot{\delta t}-c\dot{\delta t^{k}}\;\;\left[m/s\right]$$

Based on the computed ranging and power, the waveform generator generates baseband bursts $\left(d_{I}+j\;d_{Q}\right)$, with $d_{I}$ is the in-phase modulated data and $d_{Q}$ is the quadrature-phase modulated data. The baseband signal is up-converted to an intermediate frequency (IF), $F_{IF}$, then the signal is added a Gaussian thermal noise η(t) having a power spectral density No. A received Iridium burst in IF can therefore be written as:

(31) $$r\left(t\right)=\sqrt{P}\left(d_{I}\left(t+\tau \right)+j\;d_{Q}\left(t+\tau \right)\right)e^{j\left(2\pi \left(F_{IF}+F_{d}\right)t+\phi \right)}+\eta \left(t\right)$$

where $\tau =\tilde{\rho }\left(t\right)/c$ is the signal propagation time corrupted by clock errors, $F_{d}=-\dot{\tilde{\rho }}\left(t\right)/\lambda$ is the Doppler frequency shift corrupted by clock drifts, λ is the wavelength of the captured frequency channel, and ϕ(rad) is the initial carrier phase.

The baseband signal starts with a tone (i.e., $d_{I}=1,\;d_{Q}=0$) over $T_{tone}=$ 2.56 ms, followed by a BPSK word (i.e., $d_{I}=\left\{\pm 1\right\},\;d_{Q}=0$) over 1 ms, itself followed by a QPSK sequence (i.e., $d_{I}=\left\{\pm 1\right\},\;d_{Q}=\left\{\pm 1\right\}$) until the end of the burst, which is assumed to last $T_{burst}=$ 10 ms. The 25 ksps-rate data are a fake sequence of bits (the data are not used by the navigation filter). The BPSK and QPSK data bits are shaped using a root raised cosine shaping filter with a roll-off of 0.4 [2,6]. The IF signal samples are then saved to a binary file with a resolution of 8 bits.

**Figure 7:**
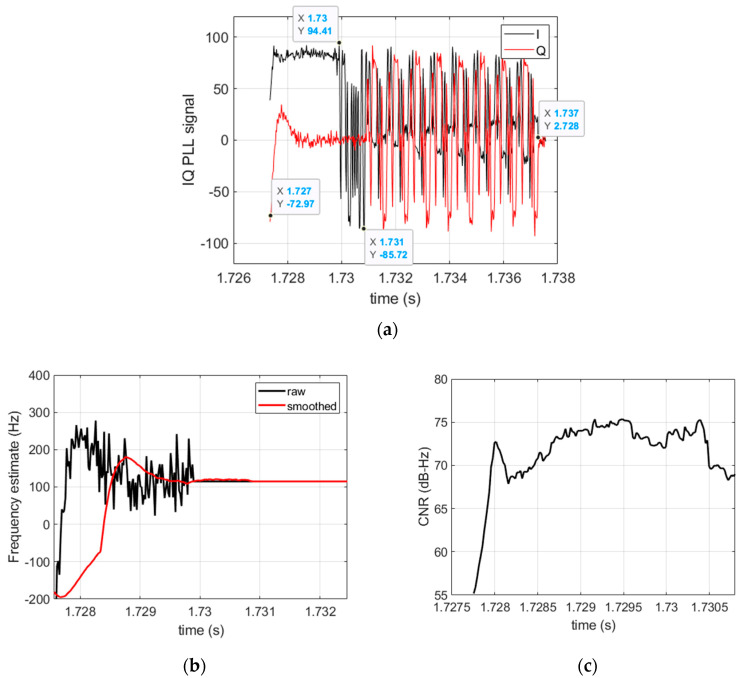
Processing results of a synthetic Iridium burst. (**a**) PLL baseband signal showing the structure of the burst; (**b**) the PLL frequency estimate; (**c**) CNR estimate.

An illustration of the processing of a generated synthetic burst by the signal processing tool is given in Figure 7. We can see the consistency of the signal structure with that of a real Iridium burst.

## 4. Performance Characterization

### 4.1. Scenario Description

The scenario consists of a high dynamic vehicle using IMU data, magnetometer data, and Iridium Doppler shift data.

#### 4.1.1. Trajectory

**Figure 8:**
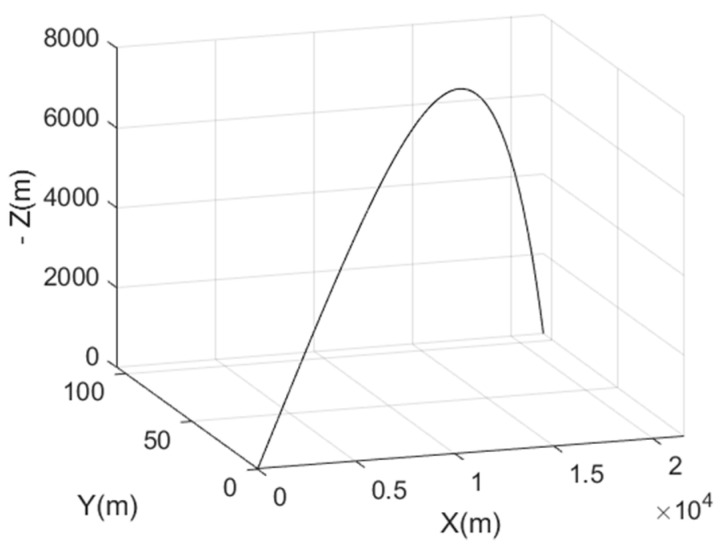
The user trajectory in the local frame.

The user trajectory (Figure 8) is generated using the Balco software [29]. It is a six- and seven-degree-of-freedom (6–7 DOF) trajectory simulation program. The user initial speed is set to 820 m/s and the initial spin rate is set to 200 rad/s. The scenario lasts 80 s, the travelled range in the *x-y* plane is ~22 km for a maximum height of ~7.8 km. We set the trajectory starting point at the latitude 75° N and the longitude 10° E in order to obtain sufficient satellite visibility for the positioning, since the Iridium satellites have polar orbits.

#### 4.1.2. Inertial and Magnetometer Data

The Balco software provides IMU and magnetometer data consistent with the six DOF trajectory of the vehicle. We used the following error model [30] to perturb the IMU and magnetometer data

(32) $$\tilde{y}=\left(I_{3}+M_{3}\right)\times y+b_{y}+n_{y}$$

where y is the 3×1 true sensor output in the body frame, $M_{3}$ is a 3 × 3 matrix that represents the misalignment error (off-diagonal terms) and sensitivity error (diagonal terms), $b_{y}$ is the 3 × 1 sensor bias and $n_{y}$ is the 3 × 1 sensor noise (assumed zero mean Gaussian). The sensors errors (1 − σ), considered of consumer-grade, are given in Table 1. We generated two hundred inertial and magnetometer data files by changing randomly the sensors error values.

#### 4.1.3. Iridium Data

The Iridium-like signal simulator of Section 3.2 is fed with the trajectory defined in Section 4.1.1 and an Iridium Next TLE file. The simulator was configured with a sampling frequency of 2 MHz and a data resolution of 8 bits. We assumed that each Iridium satellite broadcast one burst per second with a transmission power of 30 dBw. We recall that the effects of the atmosphere and multipath on signal propagation are disabled. We then generated two hundred synthetic Iridium signal files by changing randomly the bursts transmission times and the signal noise

**Figure 9:**
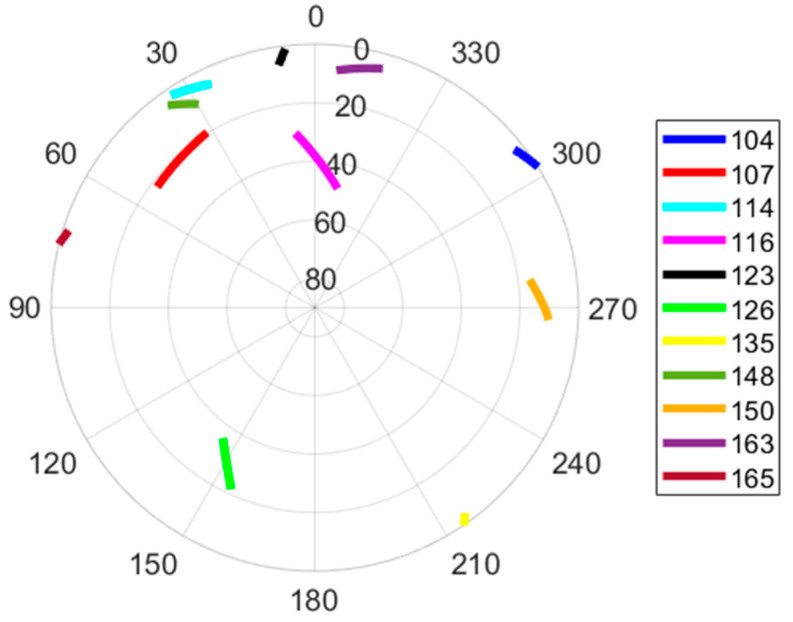
The Skyplot showing the positions of the tracked satellites in a polar projection (elevation and azimuth) centered on the user’s path. The numbers in the legend are satellite numbers.

We processed the synthetic Iridium signals by the signal processing tool. This latter was configured as in Section 3.1 (i.e., the PLL integration time is set to 10 µs, the PLL bandwidth is set to 2 kHz, and the frequency estimated by the PLL is averaged over the last 0.5 ms of the burst tone). A sky view showing the satellites positions centered on the user trajectory is given in Figure 9.

Table 2 displays the satellite availability and the received signal power. It shows that eleven satellites are captured during the scenario with an availability between 28% and 100%. In average, the number of satellites in view in any time interval of 1 s is approximately eight satellites. This satellite availability should be sufficient for the observation of the 3D position, the 3D velocity, and the receiver clock bias and drift.

### 4.2. Effect of Error Sources

The main error sources that affect the positioning using LEO SV Doppler shift measurements are the thermal noise, the satellite position and velocity errors, and the satellite clock drift. In this subsection, we study their effects on the user position and attitude determination.

We run the filter with the inertial sensors, magnetometer, and Doppler shift data described in Section 4.1. The filter states are initialized as follows. The position error is 10 km (1 − σ), the velocity error is 3 m/s (1 − σ), and the receiver clock bias error is 0.5 s (1 − σ). They are all set randomly with a Gaussian distribution. The attitude angles are randomly selected using a uniform distribution. The processing of the filter outputs permitted us to derive the performance of the filter, which will be analyzed in the following paragraphs.

#### 4.2.1. Measurement Noise

We consider the thermal noise as the unique error source (i.e., we use perfect satellite orbits and clocks). We firstly characterize the quality of the Doppler shift measurements as well as the stability of their arrival time under the effect of thermal noise.

**Figure 10:**
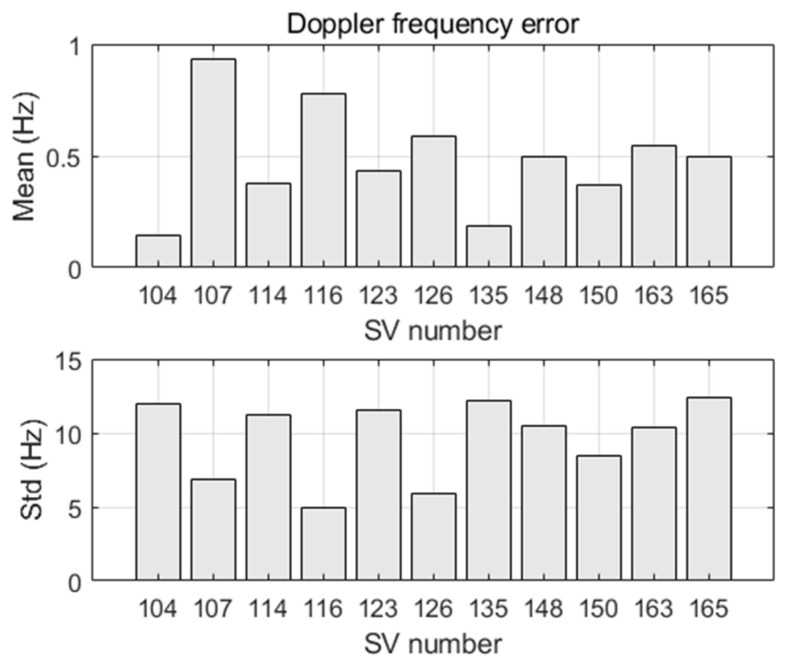
Mean and standard deviation (std) of bursts Doppler shift errors.

Figure 10 shows the statistics of the Doppler shift measurement error (here free of receiver clock drift). The mean error is smaller than 1 Hz and the standard deviation is between 5 and 12.5 Hz, which varies inversely to the satellite power level of Table 2. Despite the strong Iridium signals, the Doppler shift noise is high when compared to the GNSS Doppler noise that is typically better than 1 Hz (1 − σ). Indeed, since bursts are very short, a PLL requires a large loop bandwidth to converge within a short time, thus a small integration time that leads to high frequency noise at the PLL output. The averaging of the PLL frequency output over the last 0.5 ms of the burst tone allowed us to reduce this noise, as explained in Section 3.1.

**Figure 11:**
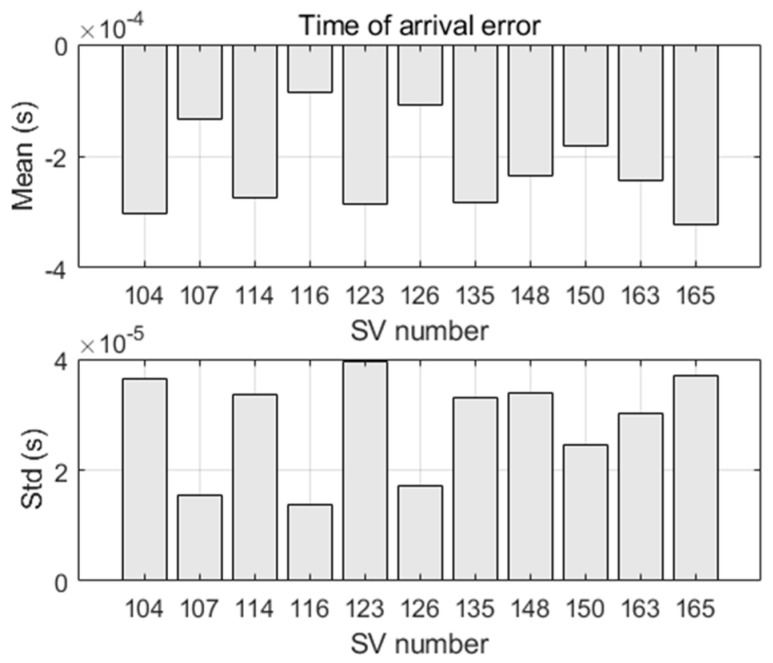
Mean and standard deviation (std) of bursts time of arrival errors.

Determining the quality of the burst time of arrival (toa) is important, since the toa is used to compute the burst transmission time (i.e., toa−τ), which is required in the calculation of the satellite position and velocity (6). Figure 11 shows the statistics of the toa error (here free of receiver clock error). The toa errors due to the thermal noise are different for the satellites. As such, they will not be compensated by the filter in the clock bias state. The toa error difference between any two satellites is smaller than 0.5 ms (mean error less than 0.4 ms, and standard deviation less than 0.04 ms).

We now discuss the performance of the navigation filter. Table 3 illustrates the root mean square error (RMSE) for the position r and attitude ε coordinates, as well as for the receiver clock bias δt and drift $\dot{\delta t}$. It shows that the IMU/magnetometer/Iridium coupling provides a positioning with an error of a few hundred meters.

**Figure 12:**
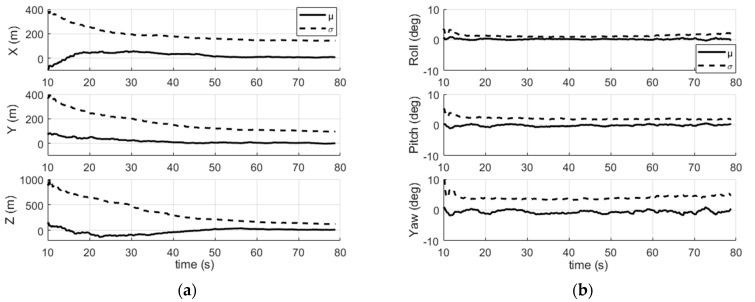

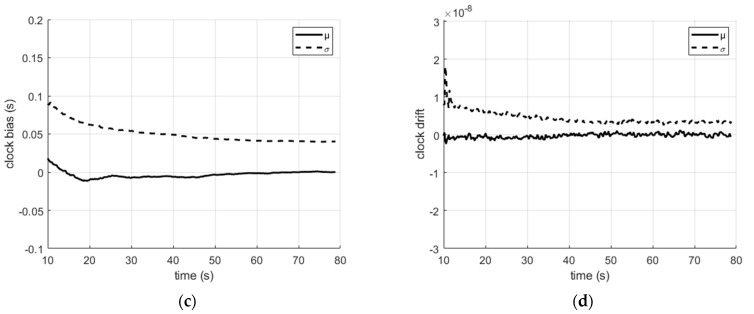
Mean (µ) and standard deviation (σ) of (**a**) the position error; (**b**) the attitude error; (**c**) the receiver clock bias error; (**d**) the receiver clock drift error. Average over 200 simulations.

In Figure 12, we can see that the filter takes a few ten seconds to converge for the position and the time states, which is the time needed to accumulate sufficient Doppler shift measurements for the state observability, and then the estimations stay stable over time.

The receiver clock bias is estimated with ~35 ms (RMSE). This is a low accuracy compared to the time correction accuracy based on pseudorange measurements in GNSS systems (typically a few ten ns). For an Iridium satellite speed of ~7.45 km/s, an error of 35 ms in burst transmission time leads to a satellite position error of ~260 m. For a positioning based on Doppler shifts, this error distorts the predicted Doppler shift. The resulting Doppler residual degrades the navigation solution.

Moreover, the scenario’s short duration combined with relatively large initial state errors has led us to increase the confidence in the Doppler measurements (despite the relatively high noise) and to enlarge the system noise matrix in order to accelerate the convergence time of the filter. Nevertheless, this happened at the expense of a degraded navigation solution. Thus, in the presence of thermal noise only, the position error is due to the Doppler shift measurement noise and the measurement time stamp errors (toa errors), and is degraded due to the scenario’s short duration.

The roll and pitch angles are estimated with an error of ~1.49° and ~1.88°, respectively, while the yaw angle error is ~4.18°. The roll is the most precise thanks to its better observation by the radial components of the magnetometer. The yaw observability is the weakest, since its value stays almost constant during the scenario. In addition, the use of magnetometer data in the observation model allowed for a faster convergence of the attitude (~15 s) than the position.

The performances presented in this paragraph, assuming perfect satellite orbits and clocks, can be considered the best we could expect from this navigation filter in the conditions of the studied scenario. They will be considered as a reference in the subsequent paragraphs to assess the effect of the other error sources.

#### 4.2.2. Satellite Position Error

The error of the satellite position computed based on TLE-SPG4 can be as high as 3 km, mostly in the tangent direction of the orbit. We study the influence of the satellite position error on the user position and attitude determination. This error is added on top of the baseline scenario that contains thermal noise. For this reason, each satellite position coordinate has a constant error added to it. Its value is randomly selected using a Gaussian distribution with a standard deviation given in Table 4. This table defines an intermediate case in which we used a typical value of the satellite position error, and a worst case in which the tangent error is increased to 3 km. It should be noted that the SPG4 model error is not Gaussian, and we assumed this distribution in order to have a random coverage of the uncertainty region of the satellite position error.

**Figure 13:**
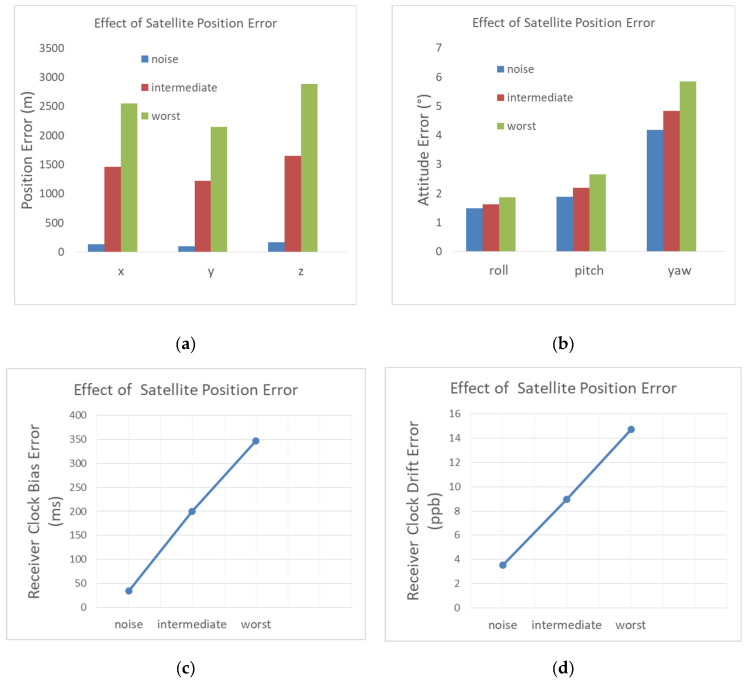
Influence of the satellite position error on (**a**) the position RMSE; (**b**) the attitude RMSE; (**c**) the receiver clock bias RMSE; (**d**) the receiver clock drift RMSE.

The navigation errors are illustrated in Figure 13 with, for comparison, those related to thermal noise that we obtained in the previous paragraph. The 3D position error rises to 2.5 km for the intermediate case of the satellite position error and it extends to 4.4 km for the worst case. The intermediate and worst satellite position errors degrade the user clock bias estimation by a few hundred milliseconds, which explains the high receiver position error.

The attitude estimation, however, is only degraded by 0.72° in the intermediate case of the satellite position error with regard to the noise only case. The attitude error degradation increases to 1.87° in the worst case. The relatively small impact of the satellite position error on the attitude determination is because in terms of the Doppler contribution, the attitude is inferred by the user acceleration variation (2), which remains small despite the satellite position error.

#### 4.2.3. Satellite Velocity Error

A satellite velocity error distorts the predicted Doppler shift and as a result affects the navigation solution estimations. This error is added on top of the baseline scenario that contains thermal noise. Each satellite velocity coordinate has a constant error added to it. Its value is selected randomly using a Gaussian distribution with the standard deviation given in Table 5. This table defines two cases, an intermediate case in which we used an intermediate velocity error, and a worst case in which the tangent velocity error is increased to 3 m/s.

**Figure 14:**
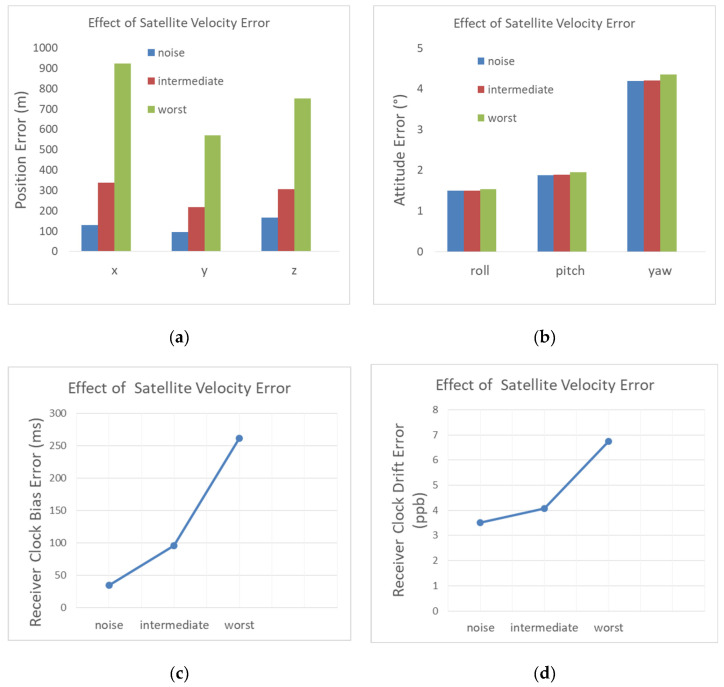
Influence of the satellite velocity error on (**a**) the position RMSE; (**b**) the attitude RMSE; (**c**) the receiver clock bias RMSE; (**d**) the receiver clock drift RMSE.

The navigation state errors are illustrated in Figure 14. For the intermediate case of the satellite velocity error, the 3D position error is degraded by 273 m with regard to the noise only case. The degradation reaches 1.09 km for the worst case. The intermediate and worst satellite velocity errors degrade the user clock bias estimation by, respectively, several tens of and a few hundred milliseconds. The attitude, however, is slightly degraded in both the intermediate and worst cases (0.03° and 0.2°, respectively).

#### 4.2.4. Satellite Clock Drift

The baseline scenario that contains thermal noise is now added constant satellites clock drifts. We set their values randomly for each satellite using a Gaussian distribution with a standard deviation set to 1 ppb for the intermediate case, and to 10 ppb for the worst case. These values can be considered as typical intermediate and worst-case values of the drift for an OCXO clock. Note that a satellite clock drift of 1 ppb (1 − σ) will add an error to the Iridium Doppler measurement of ~1.626 Hz (1 − σ). We recall that the navigation filter defined in Section 2 does not correct for this error, as it is not available in the general context of navigation based on signals of opportunity.

**Figure 15:**
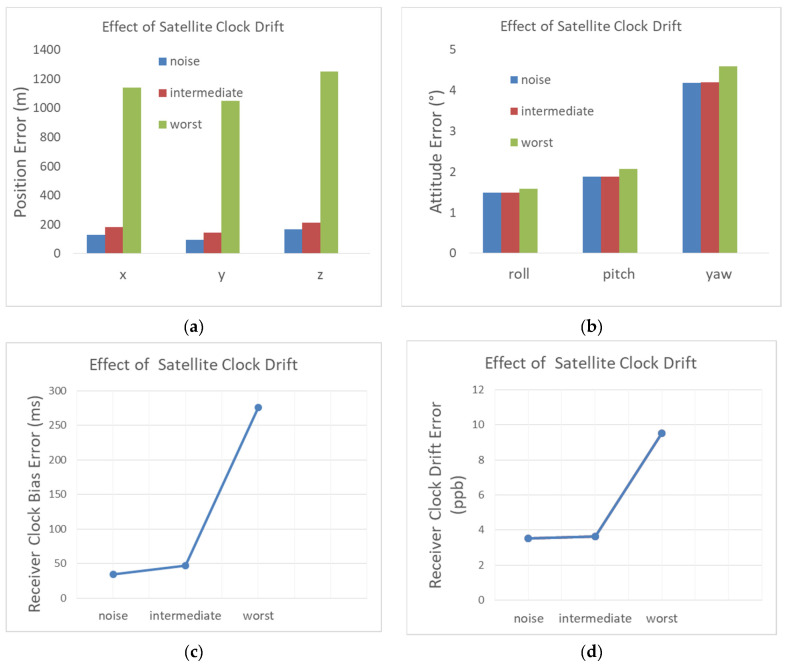
Influence of the satellite clock drift on (**a**) the position RMSE; (**b**) the attitude RMSE; (**c**) the receiver clock bias RMSE; (**d**) the receiver clock drift RMSE.

The results given in Figure 15 show that the position error is degraded by 81 m and 1.76 km, respectively, for 1 ppb (1 − σ) and 10 ppb (1 − σ) of the satellite clock drift. The intermediate and worst satellite clock drift cases degrade the user clock bias estimation by, respectively a few tens of milliseconds and a few hundred milliseconds. A satellite clock drift of 1 ppb (1 − σ) does almost not affect the attitude error, while 10 ppb (1 − σ) degrades the attitude estimation by 0.44°. This shows that in terms of the attitude estimation, a satellite clock drift of 1 ppb (order of magnitude) can be tolerated without the need for clock drift corrections.

The results obtained in this section show that the user position is highly sensitive to large satellite position errors, and to a lesser extent to the satellite velocity errors and the satellite clock drift errors. The attitude error, however, is slightly sensitive to large satellite position errors, and it is almost insensitive to the satellite velocity error and satellite clock drift.

### 4.3. Total Error Effect

Here, all of the errors are simultaneously activated with the values given in Table 6.

**Figure 16:**
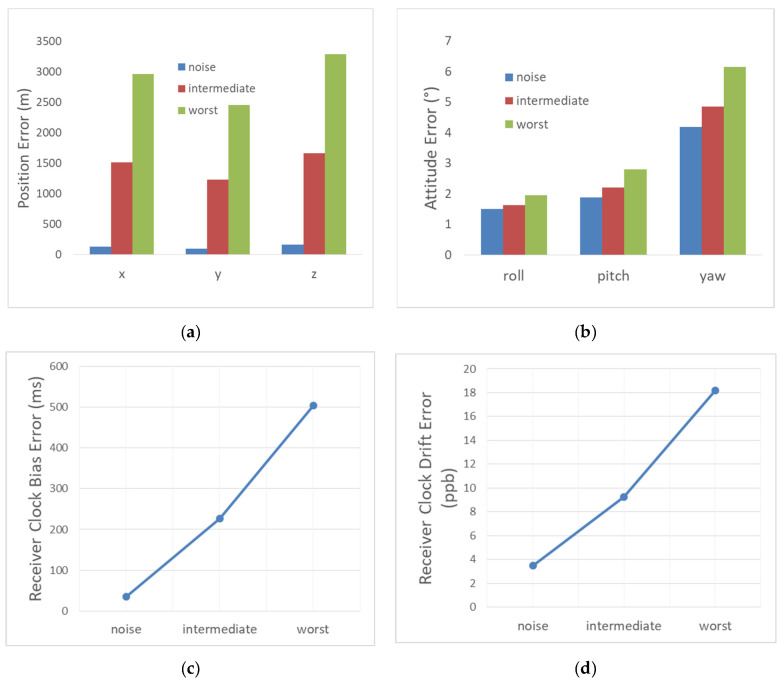
The total navigation performance. (**a**) The position RMSE; (**b**) the attitude RMSE; (**c**) the receiver clock bias RMSE; (**d**) the receiver clock drift RMSE.

The results are shown in Figure 16. The typical performance that we could expect is ~2.5 km for the position (3D), ~226 ms for the receiver clock bias, ~9 ppb for the receiver clock drift, ~1.6° for the roll angle, ~2.2° for the pitch angle, and ~4.8° for the yaw angle. The “worst” performance is ~5 km for the position (3D), ~505 ms for the receiver clock bias ~18 ppb for the receiver clock drift, ~2° for the roll angle, ~2.8° for the pitch angle, and ~6.1° for the yaw angle. We noticed that, despite the huge error sources inherent to the Iridium system used as a source of signals of opportunity for navigation, the attitude performance is relatively interesting, especially for the roll and pitch components. The convergence times of the filter states for the intermediate and worst cases are similar to that of the case using perfect satellite orbit and clock drift, mainly because we used the same initial state variance in these cases. These results assume that eight satellites are available on average during the scenario, with a measurement rate of 1 Hz, a vehicle having an initial speed of vo=820 m/s, an initial spin rate of 200 rad/s and a total scenario duration of 80 s.

### 4.4. Comparing with an INS

In many applications, the INS is typically the only backup navigation solution when the GNSS is not available. We compare the obtained position and attitude performances of the SOP-based filter (of Section 2) to those obtained with an INS based on a better quality IMU (having the specification given in Table 7), which is updated by the magnetometers of Table 1. The numerical simulations of the INS solution were carried out under the same user scenario, and by considering a perfect initialization of the position, velocity and attitude states.

The results are reported in Table 8 with, for comparison, those of the SOP-based filter obtained for best and intermediate cases of the errors. Thanks to the better IMU performance and the update by the magnetometers, the INS outperforms the SOP-based filter in terms of the attitude estimation in both best and intermediate cases, at a potentially higher cost.

On the other hand, if we assume accurate satellite orbits and satellite clock drift lower than 1 ppb, the positioning performances of the SOP-based filter are close to those of the best case (of Table 3). In this case, the positioning quality of the SOP-based filter becomes superior to the INS.

**Figure 17:**
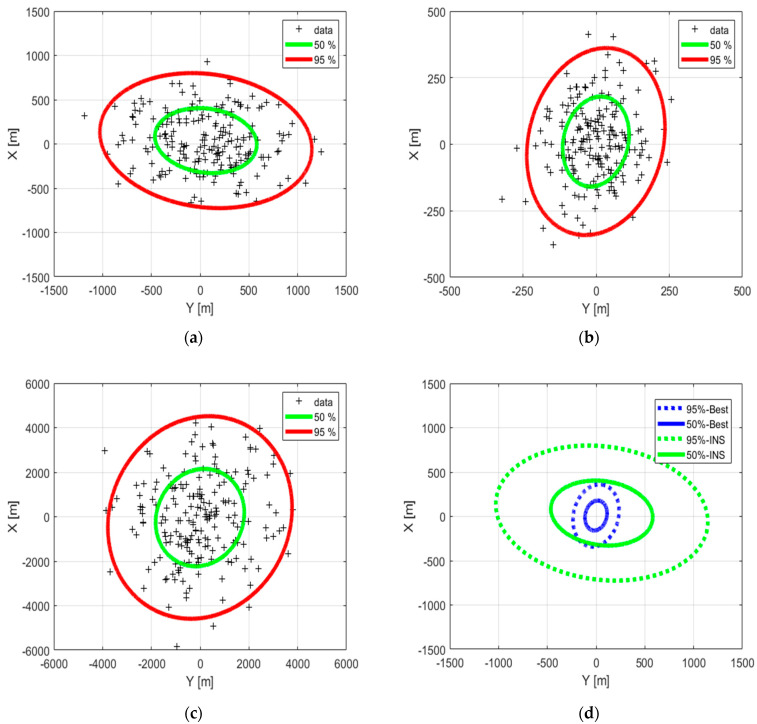
The 50% and 95% ellipses of the arrival position error. (**a**) INS; (**b**) best performance of the SOP-based filter; (**c**) intermediate performance of the SOP-based filter, (**d**) INS (green) versus best performance of the SOP-based filter (blue).

Figure 17 provides the arrival position error in the *x-y* plane (X: along range, Y: cross range) for the SOP-based filter (best and intermediate cases), as well as the INS solution. It shows that the 95% ellipse of position error of the SOP-based filter of the best case is almost inside the 50% ellipse of the INS (Figure 17d).

The Table 9 gives the semi-major (a) and the semi-minor (b) axes of the 50% and 95% ellipses of the arrival position error. We can see that the INS, based on a better quality IMU and the same magnetometers, is more accurate than the SOP-based filter using large error satellite orbits. The SOP-based filter, however, outperforms the INS solution if accurate orbits of the LEO SV are available. Furthermore, under longer scenarios its performance remains delimited over time, whereas the performance of the INS will drift more. In consequence, the filter based on LEO SV Doppler shift, consumer-grade IMU and magnetometers becomes attractive in a GNSS-denied environment.

## 5. Conclusions

In this paper, we have studied the potential of Doppler shifts of LEO SV signals for navigation, when used as signals of opportunity. We defined a filter based on the tight coupling between a consumer-grade IMU, magnetometers and Doppler shifts. The filter was fed with representative sensors data, simulated for a high dynamic vehicle. We have characterized both position and attitude performances subject to thermal noise, satellite position and velocity errors, and satellite clock drift. This allowed for assessing and understanding the contribution of each error source to the navigation error. We have shown that, while the positioning error is large due to large orbit errors or high SV clock drifts, it becomes competitive with that of an INS based on a better quality IMU, if precise satellite orbits are available.

Contrary to the positioning, which is highly affected by large error sources, we have shown that the attitude estimation is less sensitive to large orbit errors and high SV clock drifts. For typical values of the error sources (the intermediate case), the defined SOP-based filter, which uses the SPG4 model fed with the broadcast NORAD TLE files, is able to track the vehicle’s attitude with a roll angle RMSE of ~1.6°, a pitch angle RMSE of ~2.2°, and a yaw angle RMSE of ~4.8°. These results illustrate the potential of the LEO SV signals of opportunity for the navigation of high dynamic vehicles in a GNSS-denied environment. Recently, the European Union (EU) announced that it will launch its own system of satellite-based high-speed internet [31]. Since large satellite orbit errors and clock drifts are the main factors limiting the performance of the Doppler shift-based positioning, if such a system provides accurate satellite positions and clock drifts, then it could in addition offer a navigation service that will be very useful in GNSS-denied environments.

## Figures and Tables

**Table 1:** A consumer-grade sensor error specification (1 − σ).

	Accelerometer	Gyroscope	Magnetometer
Noise	0.1 m/s/√ (h)	3.6 deg/√ (h)	0.2 mG/√ (hz)
Bias	50 mg (0.5 m/s^2^)	1000 deg/h	1 mG
Sensitivity	2000 ppm	2000 ppm	2000 ppm
Misalignment	0.1 deg	0.1 deg	0.1 deg

**Table 2:** Satellites availability and received bursts power (averaged over 200 simulations).

SV numbers										
104	107	114	116	123	126	135	148	150	163	165
Satellite availability (%)										
36	100	96.6	100	99.7	100	28.8	73.7	100	100	51.1
Received power (dBw)										
−136	−131.1	−135.5	−128.1	−135.7	−129.7	−136.1	−134.6	−133.0	−134.7	−136.3

**Table 3:** Root mean square error for position, attitude and time.

	X	Y	Z
Δr (m)	128.84	95.90	166.60
	Roll	Pitch	Yaw
Δε (deg)	1.49	1.88	4.18
Δ(δt) (ms)	34.68		
$\Delta \dot{\left(\delta t\right)}$ (ppb)	3.51		

**Table 4:** Standard deviation of the satellite position error.

	Radial	Tangent	Normal
Intermediate case	300 m	1700 m	150 m
Worst case	300 m	3000 m	150 m

**Table 5:** Standard deviation of the satellite velocity error.

	Radial	Tangent	Normal
Intermediate	0.3 m/s	1 m/s	0.15 m/s
Worst	0.3 m/s	3 m/s	0.15 m/s

**Table 6:** Error sources definition (1 − σ) for an intermediate case and a worst case.

Error Source	Intermediate	Worst
Satellite clock drift	1 ppb	10 ppb
Satellite tangent position error	1700 m	3000 m
Satellite tangent velocity error	1 m/s	3 m/s

**Table 7:** A tactical-grade sensor error specification (1 − σ).

	Accelerometer	Gyroscope
Noise	0.1 m/s/√ (h)	0.25 deg/√ (h)
Bias	10 mg (0.1 m/s^2^)	50 deg/h
Sensitivity	1700 ppm	1000 ppm
Misalignment	0.1 deg	0.1 deg

**Table 8:** Root mean square error for position and attitude, comparing the SOP-based filter with an INS based on a tactical-grade IMU and magnetometers.

	X	Y	Z
Δr (m)—INS	181.78	238.02	182.312
Δr (m)—SOP-based (best)	128.84	95.90	166.60
Δr (m)—SOP-based (intermediate)	1512.89	1232.75	1666.89
	Roll	Pitch	Yaw
Δε (deg)—INS	0.45	0.27	0.63
Δε (deg)—SOP-based (best)	1.49	1.88	4.18
Δε (deg)—SOP-based (intermediate)	1.63	2.20	4.85

**Table 9:** Semi-major (a) and semi-minor (b) axes of the 50% and 95% ellipses of arrival position error.

	INS	SOP-Based	SOP-Based
		(Best)	(Intermediate)
a (50%) (m)	526	170	2211
b (50%) (m)	360	111	1790
a (95%) (m)	1095	354	4597
b (95%) (m)	750	231	3722

## Data Availability

Not applicable.
